# Trade-offs in motivating volunteer effort: Experimental evidence on voluntary contributions to science

**DOI:** 10.1371/journal.pone.0224946

**Published:** 2019-11-21

**Authors:** Elizabeth Lyons, Laurina Zhang

**Affiliations:** 1 School of Global Policy & Strategy, University of California, San Diego, La Jolla, CA, United States of America; 2 Scheller College of Business, Georgia Institute of Technology 800 W Peachtree St NW, Atlanta, GA, United States of America; Iowa State University, UNITED STATES

## Abstract

Digitization has facilitated the proliferation of crowd science by lowering the cost of finding individuals with the willingness to participate in science without pay. However, the factors that influence participation and the outcomes of voluntary participation are unclear. We report two findings from a field experiment on the world’s largest crowd science platform that tests how voluntary contributions to science are affected by providing clarifying information on either the desired outcome of a scientific task or the labor requirements for completing the task. First, there is significant heterogeneity in the motivations and ability of contributors to crowd science. Second, both of the information interventions lead to significant decreases in the quantity and increases in the quality of contributions. Combined, our findings are consistent with the information interventions improving match quality between the task and the volunteer. Our findings suggest that science can be democratized by engaging individuals with varying skill levels and motivations with small changes in the information provided to participants.

## Introduction

Digitization has facilitated the proliferation of crowd science, where the general public is voluntarily engaged in scientific research activities. While the concept of crowd science is not new, the number and type of crowd science projects have sharply increased in the past decade. The Internet has lowered the cost of finding individuals with the ability to participate in scientific activities at different stages of the research process and technology has made it easier to aggregate and compare a large quantity of data from different participants, thus improving the accuracy and reliability of crowd science contributions. Examples of digital crowd science projects include classifying images of galaxies (Galaxy Zoo), modifying the shape of a visual 3D model of a protein to optimize its shape (Foldit), using smartphone games for neurology research [1], and finding solutions to prevent and treat tuberculosis [2]. While these activities have been shown to be economically important [3], the non-pecuniary incentives that motivate volunteer participation and performance are less understood.

Importantly, the effectiveness of non-pecuniary incentives for volunteers in science depend on understanding volunteer motivations and abilities [4]. If volunteers are heterogeneous in their beliefs about the value of their contributions to a task, information that clarifies the eventual outcome of a task or the type and amount of labor required for a task can generate positive belief updating among individuals whose priors lead them to incorrectly believe they were ill matched for the task and negative updating among individuals whose priors lead them to incorrectly believe they were well-matched for the task [5]. We argue that providing volunteers with more information about the value of their labor output or input have theoretically ambiguous impacts on effort in terms of the quantity and quality of their contributions because it is unclear ex-ante what individuals’ beliefs are in the absence of this information. We examine whether the quantity and quality of voluntary contributions are affected by two information treatments. We manipulate whether participants receive information on 1) the specific project outcome that volunteer labor will contribute to in order to improve volunteer matching based on the project mission [6], and 2) the specific labor input requirements of the task [4] in order to improve volunteer matching based on skill needs. This study is the first to examine whether motivation based on project mission or labor requirements affect the quantity and quality of outcomes. We do so using a randomized control trial (RCT) on the world’s largest crowd science platform.

We document two key findings. First, both information treatments improve the quality (i.e., accuracy) of contributions. Second, however, the treatments reduce the quantity of contributions completed per participant without changing the total time participants spend on the task. While effort on our task is difficult to measure precisely, we consider the distinction between effort invested in quantity and quality as distinct and important dimensions of this measure. Effort spent on classifying a large number of images without attention to accuracy is unlikely to be difficult, as it only requires mouse clicks. Accurate image classification is reasonably challenging and requires some amount of focus both on the questions being asked and the image being presented [7]. Therefore, we interpret the higher accuracy among the treatment groups to indicate they were more willing to spend effort on the more difficult aspect of the task than those in the control. This increased willingness to undertake a costly action is consistent with volunteers in the treatment groups being more intrinsically motivated by the task than those in the control. We also find suggestive evidence that the treatments had meaningful impacts on contributor motivations and backgrounds, and, thus, that they affected the types of people who contributed to the task. Our results show that minor clarifications in science task objectives can induce significant changes in the performance of contributors, potentially by changing the types of people who contribute, even for tasks that require relatively low skill.

## The Zooniverse experiment

We investigate our research question by running a RCT on Zooniverse, the world’s largest crowd science platform. Scientists post projects in a variety of fields (e.g., art, astronomy, biology, history, etc.) on Zooniverse that require contributors to answer questions about images the scientists would like to classify. Over 1 million registered Zooniverse contributors assist in these projects with the understanding that they will not be paid or formally acknowledged for their contributions. One notable feature that makes Zooniverse an attractive setting for isolating the impacts of task framing on outcomes is that contributors are anonymous and do not see each other’s contributions. In addition, contributors rarely interact with each other on the platform, which suggests that reputation and social returns are unlikely motivations for contributions in this setting.

Responses to a survey administered by Zooniverse demonstrate that the majority of contributors have at least a Bachelor’s degree and over 25% have a graduate degree. In addition, the majority are employed in some capacity suggesting they have outside options that pay wages. Over 60% of contributors who responded to the survey reside in the United States or the United Kingdom.

### Contribution task and experimental treatments

The project we posted on Zooniverse is part of a larger East African rangeland crowdsourcing project that seeks to improve interventions aimed at assisting pastoralists in the face of increasing drought risks. During their routine herding, participating pastoralists took pictures of rangelands in Northern Kenya and completed a survey in which they classified the types of vegetation in the photos. For more information about this project, see https://www.udiscover.it/applications/pastoralism/. We used a subset of these photos for our study classification task.

The task involves classifying 1,061 images on six dimensions: 1) Is there any green grass? 2) Are there trees? 3) Are there shrubs? 4) Is this a picture of a rangeland? 5) Is this picture poor quality (i.e., blurry, poor lighting or angle)? 6) Are there wild/domesticated animals?

**Fig 1:**
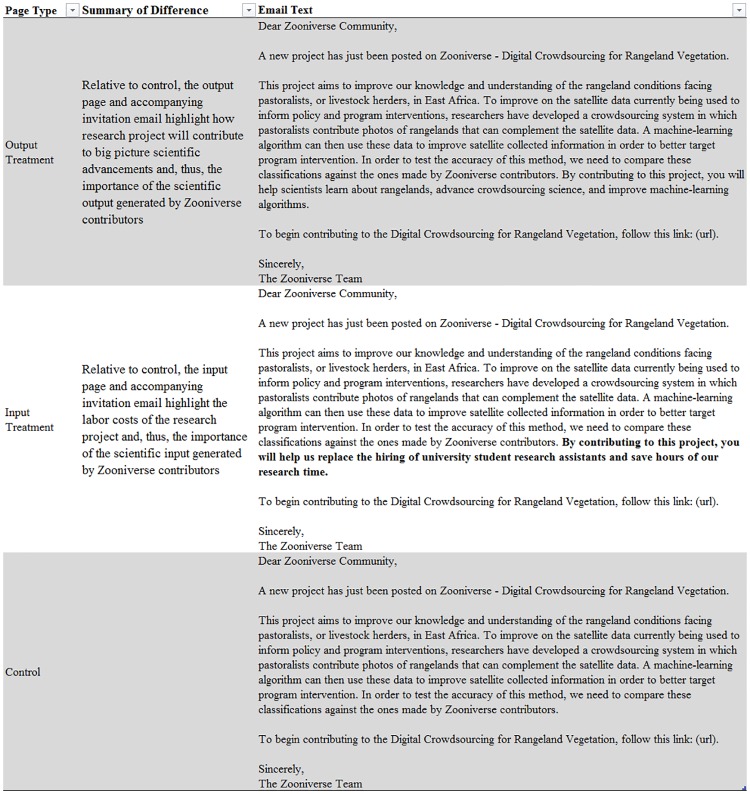
Summary of treatments relative to control. Figure summarizes the differences between the output treatment, the input treatment, and the control. Bold text is text not included in other email scripts. The complete text provided in all three project pages are presented in the text displayed in S2 Fig.

We generated three separate project pages on Zooniverse with the same scientific content and background information that differed only in their emphasis on the value of the individual’s voluntary labor output and input, respectively. All three project pages asked contributors to classify vegetation and wildlife in images of African rangelands. On the output treatment page, we emphasized how volunteer effort would contribute to an eventual project outcome on the email invitation, Zooniverse landing page, and project description page. On the input treatment page, we emphasized the value of contributors’ labor supply and skill requirements on the email invitation, Zooniverse landing page, and project description page. The control page did not include separate text related to the value of contributors’ output or input. A summary of the differences between the input and output pages relative to the control is provided in Fig 1. The additional text included on the input and output pages totaled one sentence on the email invitation and on the landing and project description phases respectively relative to the control page. This “light touch” intervention is employed to minimize concerns about potential contributors selecting in or out of a page based on a higher volume of text alone. The full text displayed on the project pages are provided in text displayed in S1 Fig for this article.

All three pages also included identical invitations for contributors to complete a short, anonymous survey about themselves and their motivations for contributing. This invitation was provided in a banner displayed on every tab and stated “In an effort to better understand who our contributors are and how we might be able to attract more, please fill out a short, anonymous questionnaire at the top right corner of our project page.” Although project pages generally do not include a contributor questionnaire, after consulting with the Zooniverse team on the issue, we concluded that including one was unlikely to be considered inappropriate or disconcerting by contributors. Moreover, it was Zooniverse’s preferred method for us to collect survey responses.

### Implementation

In order to test whether the treatments have an effect on page contributions, we need to ensure that all three pages were viewed by comparable populations of contributors. Ideally, we would show the three pages to a set of contributors and have them choose which page to contribute to based on their motivations and preferences. However, this was not possible because it would have alerted contributors to the experiment and led to potential bias in our estimates [8]. To address these concerns, we worked with the Zooniverse organization to keep the project pages private from the general population of contributors and allow only a randomly selected 1,100 contributors to access each page through a link provided in an email sent by the official Zooniverse email account. Given the large number of total email recipients, and that the recipients were randomly assigned across 3,300 contributors, we are confident that the average email recipient is statistically the same across the three pages.

The email text inviting people to make contributions to the pages was identical in all three cases except for the inclusion of the statement “Your contributions will help the advancement of crowd science and machine-learning algorithms!” in the output page treatment email, and the inclusion of the statement “Your contributions will help us replace the hiring of university student research assistants and save hours of our research time!” in the input page treatment email. In addition, the three pages had slightly different titles because the platform does not allow separate pages to have the same title. Specifically, the title of the control page project was “Crowdsourcing for Rangeland Vegetation”, the title of the output page project was “Digital Crowdsourcing for Rangeland Vegetation”, and the title of the input page project was “Digital Crowdsourcing for Vegetation”. The full email text is provided in the text displayed in S1 Fig.

While we would like to separate the effect of the task framing on selection into contributing from the effect of task framing on the effort exerted among those who do contribute, the nature of the setting does not allow us to do so. Respondents can choose not to participate in the task even after clicking on the email link, and we are not able to observe the characteristics of these people that select out of participating. As such, we are unable to disentangle the extent to which differences in the quality and quantity of work performed across our three pages are driven by differences in who contributes or by differences in how contributors contribute. We interpret our results as the total impact of task framing on outcome selection, combining its impact on selection into the task (i.e., who participates) and selection into effort conditional on performing the task (i.e., how well they participate).

This study was approved by the UC San Diego Institutional Review Board, project number 161424. Study participants were not aware they were participating in the research study reported in this paper. A waiver of consent was approved for this study because the research could not be practicably implemented without the waiver. The waiver was requested primarily to ensure contributors were responding to the interventions as they would for other Zooniverse posted projects, and not to our experiment [8].

### Data and analysis

Our final sample includes 197 participants across all three pages (61 control, 72 output, 64 input). A total of 201 contributors were observed opening the classification task, but 4 of these did not log their contributions. These sample sizes are comparable to the sample size in related studies that analyze the decision to donate time to charitable causes [9, 10]. We have also assessed whether the response rate of our project is in line with other Zooniverse projects by dividing the average number contributors in a Zooniverse project [3] by the total number of registered Zooniverse users. These numbers give us an average response rate of less than 2%, which suggests that our response rate is slightly higher than the expected response rate on this platform. These response rates are also consistent with voluntary participation rates on other crowd-based platforms. For instance, Wikipedia has over 37 million registered users that help edit the pages but only about 123,000 contribute regularly (0.3%), and even less users actively participate in community discussions and steward elections. Data on contributor characteristics, including education, income, employment status, and Zooniverse activity was collected from the subset of contributors who responded to the anonymous contributor questionnaire posted on the project pages. The full set of survey questions is provided in S3 Fig. The survey response rate was 48% for the control page, 44% for the output page, and 39% for the input page.

We examine contributor effort both by the number of classifications they complete, and by the quality, or accuracy, of their completed classifications. The quantity of classifications per contributor, and the classifications made were collected from Zooniverse. These data are provided to all researchers who posted projects on the platform, and our data collection method complied with the terms and conditions of the website.

To generate a measure of classification accuracy on which to compare contributor classifications, both authors separately classified all 1,061 images. In addition, we used data from the pastoralists’ classifications on grass, shrub, and trees. Pastoralists did not classify whether there were animals in the photos. Moreover, they were required to submit high quality photos of rangelands so they were not asked whether their photos were of rangelands are of poor quality. Both of these baseline accuracy measures are useful for different reasons. Pastoralists have expert knowledge of the rangelands in the project images and, therefore, their classifications are based on better knowledge of the rangelands than those completed by the authors. The authors’ classification data also includes information on image quality, the presence of animals, and whether or not the image is of a rangeland. Our preferred measure of question accuracy restricts the sample of image-question observations to those for which both authors and the pastoralist agree in the case of grass, trees, and shrubs, and to those for which both authors agree for the remaining questions, and drops image-questions for which there does not appear to be a clear answer. However, our findings are robust to less stringent measures of accuracy, such as comparing classifications only against those of pastoralists.

With this measure of image-question accuracy, we generate a combined accuracy measure that equals one if each question of the image was answered accurately, and zero otherwise. Using this quality measure at the image level of observation, we generate our indicator of quality, which is a count of the number of high quality classifications made by each contributor weighted by the proportion of contributor classifications that are included in the combined image accuracy measure. The dataset used in our analysis is publicly available through UC San Diego’s Digital Collections data repository [11]

## Results

**Fig 2:**
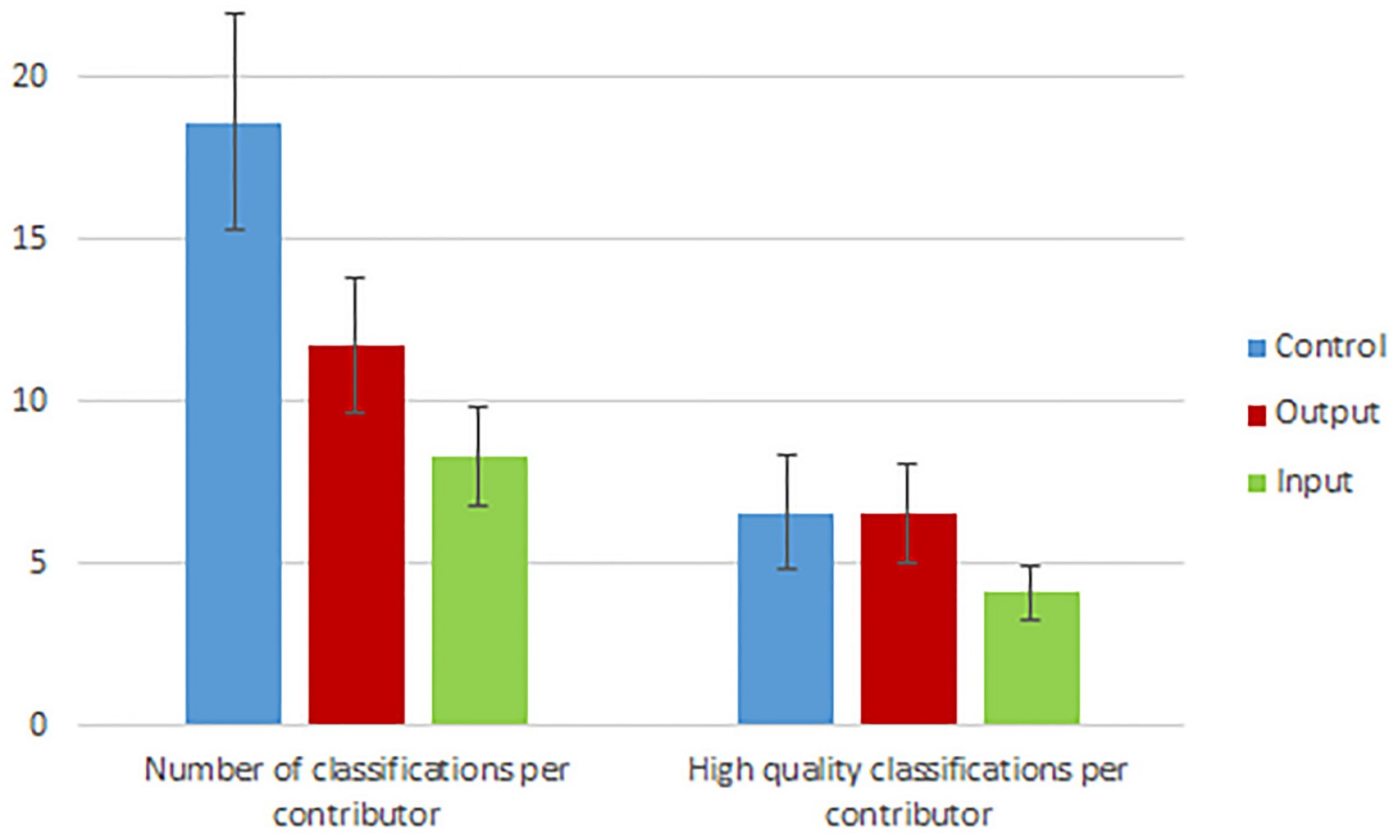
Contribution quantity and quality by treatment groups. Figure plots the average quantity and quality of image classifications on Zooniverse across control and treatment pages along with standard errors. Because not all images are assessed for quality, we weight by the proportion of images that are in our quality weighted quantity measure.

We find that information treatments that emphasize output or input affect the quantity and quality of contributions. While the number of contributors to each page is statistically the same, significantly more images were classified on the control page than the output and input pages. In fact, as Fig 2 demonstrates, control participants classify more than double the number of images than input participants. However, once we weight the number of classifications by quality, the number of classifications are not statistically different across pages (Fig 2). This is because the input and output treatments are associated with more accuracy on classification questions. In particular, 49% of output classifications and 45% of input classifications are accurate across all task dimensions compared to 35% of classifications on the control page (p-values of difference relative to control are 0.03 and 0.155 respectively). The finding that the output treatment leads to significantly higher classification accuracy is robust to controlling for image fixed effects in a regression (see S1 Table).

It is worth noting that, although the difference between the quality weighted quantity of output generated by the input and control pages is statistically the same in our sample, power analysis demonstrates that with our sample means and standard deviations, the control page quality weighted quantity would become significantly higher with 260 contributors per page which most pages on Zooniverse surpass.

**Fig 3:**
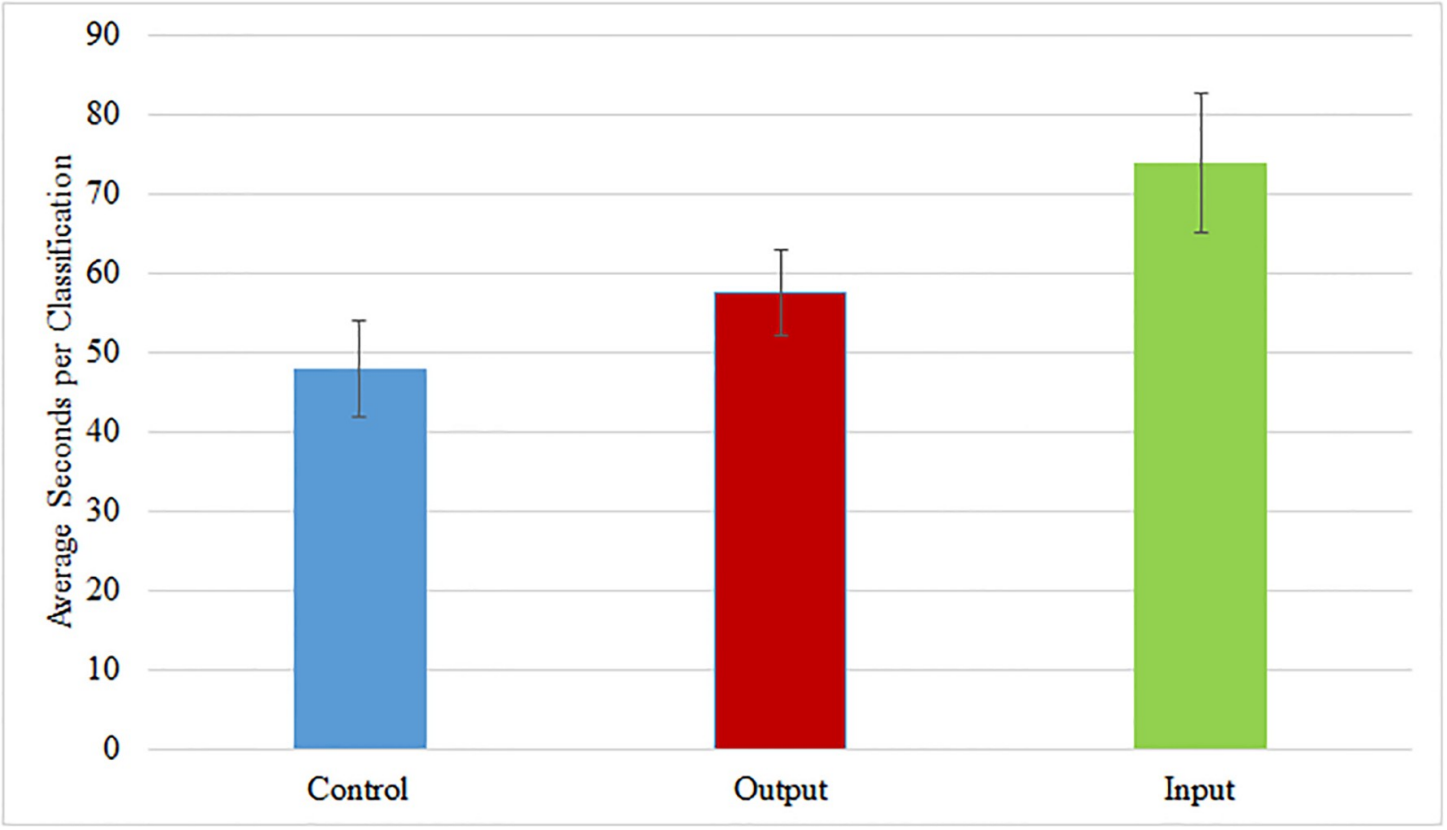
Average time spent in seconds per classification by treatment groups. Figure plots the average number of seconds contributors spend on a single image classifications on Zooniverse across control and treatment pages along with standard errors. Sample excludes contributors who spent over 6 minutes per classification on average as these likely represent contributors who left Zooniverse open while inactive on the site.

We also find that input contributors spend significantly more time per classification compared to control contributors (Fig 3). This result holds when outliers, defined as participants who spend 6 minutes or more per classification on average, are dropped. We believe dropping these participants is important because the length of time per classification indicates they may be leaving their browsers open while performing other tasks or activities and, thus, falsely driving up the average time spent on the project. This is consistent with a quantity-quality trade-off between the control and treatment pages, where contributors to the treatment pages appear to exert more effort per classification in terms of accuracy at the expense of completing a fewer number of classifications. Given that accuracy requires contributors to pay attention to the questions being asked and to the image characteristics and quantity simply requires a series of mouse clicks, these findings demonstrate that contributors to the treatment pages were more willing to spend effort on the more difficult aspects of the task than those who contributed to the control page.

**Fig 4:**
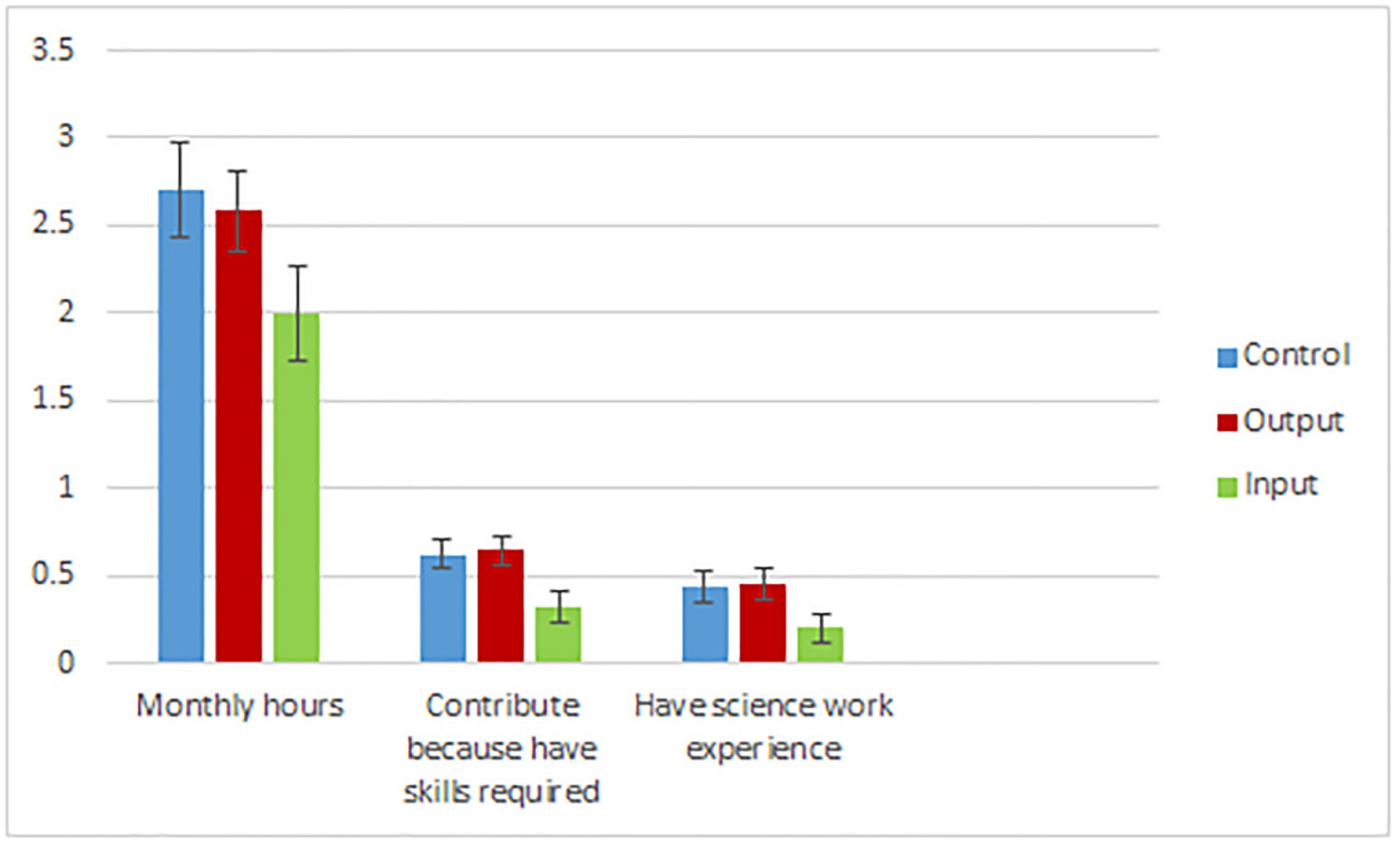
Contributor characteristics by treatment groups. Figure plots the average Zooniverse contributor characteristics across control and treatment pages along with standard errors.

As we discuss above, these differences in the willingness of contributors to exert effort on the more difficult aspects of the task across treatment and control could be driven by changes in motivations among individuals who would always contribute, changes in the types of people who contribute, or both. Given that contributors self-select into the task, our sample of contributors are likely all very motivated. However, even among this highly motivated sample, the possibility that the treatments alter how motivated contributors are is supported by existing evidence. For instance, even among charity donors who donate similar amounts, there is significant variation preferences for the effectiveness of the organizations they donate to [12]. While we cannot conclusively disentangle changes in motivations among existing contributors from changes in the types of people who contribute, evidence from our contributor survey provides suggestive evidence that the treatment effects on outcomes are driven at least in part by differences in the pool of contributors across pages. In particular, Fig 4 demonstrates that contributors who completed the survey on the control page claim to spend the most time per month contributing to Zooniverse projects (7-10 hours), significantly more than the time spent by input page contributors (3-6 hours). Moreover, while almost all contributors indicate they contribute to projects on Zooniverse because they like that they are contributing to science both output and control contributors who completed the survey are significantly more likely to indicate that they contribute because of their skill-set than input page contributors. Consistent with this, both output and control contributors in the survey sample are significantly more likely to indicate that they have science related work experience. The three groups do not differ in their average education level, employment status, age, or income.

**Fig 5:**
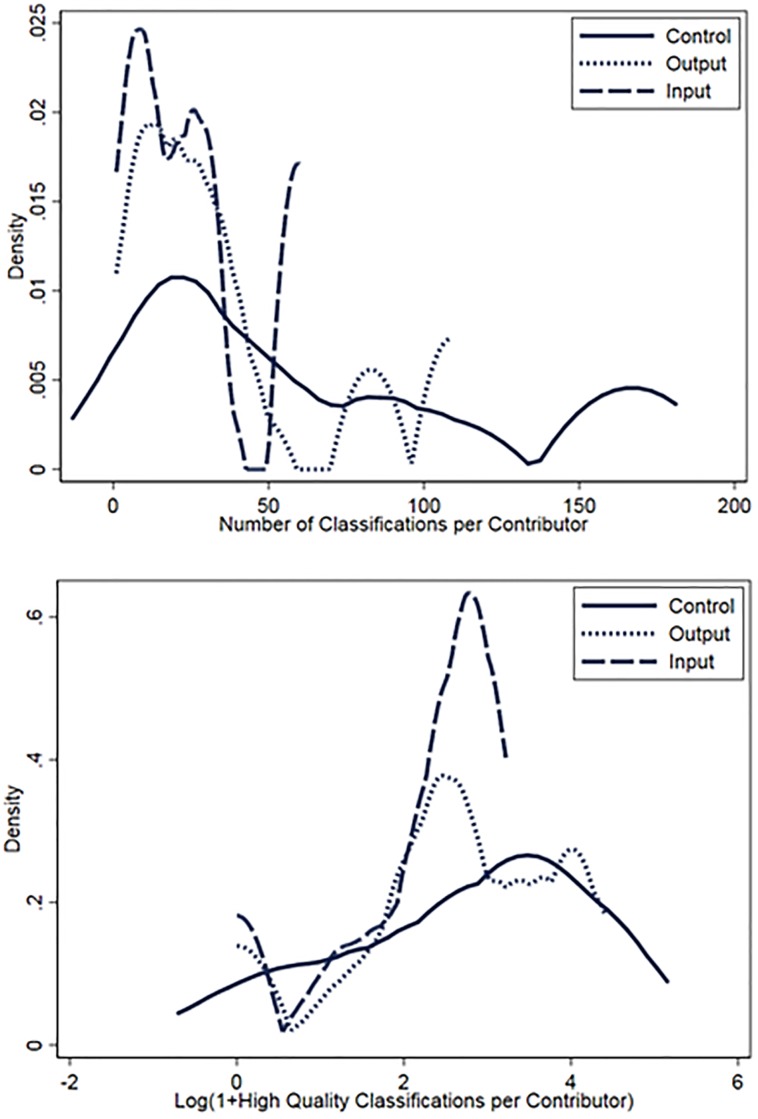
Kernel densities of contributor quantity and quality by treatment groups. Figure plots the Epanechnikov kernel density of the number of classifications completed per user (Panel A) and number of high quality classifications completed per user (Panel B) by treatment group. The narrower distribution plot for the treatment lines suggest that the probability of an outcome (i.e., the number of classifications completed per contributor or the number of high quality classifications) on treatment pages falls under a tighter band compared to the control.

The sample of contributors who responded to our survey is a subset of all contributors. Since we cannot observe the characteristics of those who did not respond to our survey, we cannot be certain about how representative these responses we did receive are of our sample population. Therefore, the patterns presented in Fig 4 should be considered suggestive rather than conclusive. However, to provide further evidence of this potential self-selection effect, kernel density plots (Fig 5) demonstrate that contributors to the treatment pages are more homogeneous in their contribution patterns (i.e., less disperse) relative to the control. The narrower distribution plots for the treatments suggest that the probability of an outcome falls under a tighter band compared to the control, suggesting that the treatments led to convergence in beliefs about the value of contributor output and input.

Combined, our findings indicate that information that clarifies input requirements and output values of volunteer research tasks impacts the quality and quantity of contributions. This finding is consistent with the interpretation that the information treatments are associated with better matching on individuals’ skills and motivations. This improved matching is associated with more cognitive control in the form of attention invested in the task [7] which improves accuracy at the expense of a lower number of classifications completed. While prior literature tended to focus on the extent to which providing information on the mission of an organization can affect outcomes [13–15], our results suggest that clarifying labor requirements can also affect participation and performance outcomes. It also provides an additional channel through which science can be democratized. In addition, our results that suggest improved information on skill requirements may improve matching on volunteer capabilities is consistent with existing evidence from paid labor opportunities [16], but to our knowledge, has previously not been studied in the context of volunteer labor. Our results underscore the fundamental trade-off between quality and quantity of production workers [17] and shows that it remains an important consideration on digital platforms for volunteers, where attracting large quantities of volunteers is relatively easy but screening volunteer quality is difficult.

## Implications

Our study confirms that even within the subset of individuals who select into participation on science-based crowdsourcing platforms like Zooniverse, there is substantial heterogeneity in volunteer motivations and skill sets. These differences have implications for how successfully volunteer labor can contribute to research progress. Importantly, since there is substantial heterogeneity in the types of research tasks that seek voluntary contributions, matching volunteer types to project objectives has the potential to meaningfully improve research outcomes. For instance, some routine tasks require a large number of participants to generate a solution due to the sample size requirements of some algorithms [18], while more complex and uncertain tasks require knowledge diversity and specialized skills [19]. Crowd science tasks vary across these dimensions, with some being routine, well-understood tasks that can be broken into a series of linear steps that include a defined range of acceptable solutions (e.g., tagging images, improving search results), and others being non-routine, complex tasks (e.g., generating product ideas, solving complex problems) that can be approached in different ways [20]. Researchers posting the former type of project may benefit from providing less information on why volunteers are being sought in order to maximize the quantity of contributions by reducing volunteers’ attachment to the project and thus, the time they spend per task element. In contrast, researchers posting the latter type of project may benefit from providing specific information on the types of skills and motivations they are looking for to ensure volunteers who select into the task invest in performing high quality output.

In addition, our findings demonstrate that science can be democratized by engaging individuals with varying skill levels. We show that even in a setting where the skill-level required for a task is low, small changes in the information provided can facilitate participation by individuals who may otherwise underestimate their ability to contribute to science. This suggests that, to the extent that breaking down large scale and complex scientific projects into smaller and simpler pieces is possible, inducing the engagement of non-scientists in the research community can have a meaningful aggregate impact on overall science output.[21].

## Supporting information

S1 Table Effect of output and input treatments on accuracy. This Table compares how the treatments affect the accuracy of classifications within the same image. Robust standard errors are in parentheses. All columns include image fixed effects. * significant at 10%; ** significant at 5%; *** significant at 1%.
(TEX)

S1 Fig Project page text. The Figure displays the text on the output treatment, input treatment, and control project pages. The text displayed in italics is only included on the input page, and text underlined is only included on the output page.
(TEX)

S2 Fig Invitation email text. The Figure displays the text in the output treatment, input treatment, and control invitation emails. The text displayed in italics is only included on the input page, and text underlined is only included on the output page.
(TEX)

S3 Fig Contributor survey. The Figure displays the survey text and questions given to contributors who opted to complete it.
(PDF)
