# Supplementary material for: Trade-offs in motivating volunteer effort: Experimental evidence on voluntary contributions to science
Source: PLoS One. 2019 Nov 21;14(11):e0224946. doi: 10.1371/journal.pone.0224946 (PMC6871885; doi:10.1371/journal.pone.0224946)
Supplement: S3 Fig — The Figure displays the survey text and questions given to contributors who opted to complete it. (PDF) [file pone.0224946.s004.pdf]

Thank you for your interest in our project on Rangeland Conditions in Northern Kenya. In order to develop a better understanding of why people are contributing to our project, we are asking all our contributors to provide some information about themselves by filling out this 8 question survey. We expect that completing the questions will take you no more than 5 minutes. Thank you!

* Required

1. **1. On average, how many hours per month do you spend contributing to Zooniverse projects?** *

Mark only one oval.

- ◯ 0-2
- ◯ 3-6
- ◯ 7-10
- ◯ 11-20
- ◯ 21 or more
- ◯ Prefer not to answer

2. **2. Why do you contribute to projects on Zooniverse? Check all that apply.** *

Check all that apply.

- ☐ The projects are fun
- ☐ It's a good way to pass the time
- ☐ I like that I am contributing to science
- ☐ I have a background in science and want to make use of it
- ☐ I am good at classifying items on Zooniverse
- ☐ Prefer not to answer
- ☐ Other: ____________________

3. **3. Have you studied or worked in a social or natural science-related field? Check all that apply.** *

Check all that apply.

- ☐ I am taking or have taken a course in science
- ☐ I am majoring or have majored in a science-related discipline in school
- ☐ I am working or have worked in a science-related field
- ☐ I have experience with science-related fields in other ways
- ☐ Prefer not to answer
- ☐ Other: ____________________

4. **4. What is the highest degree or level of school you have completed? If currently enrolled, highest degree received.** *
Mark only one oval.

- Some high school, no diploma
- High school graduate, diploma or equivalent (e.g. GED)
- Some college, no degree
- Trade/technical/vocational training
- Bachelor's Degree
- Master's Degree
- Doctorate, law, or medical degree
- Prefer not to answer

5. **5. What is your current employment status?** *
Mark only one oval.

- Employed for income full-time
- Employed for income part-time (less than 25 hours/week)
- Self-employed
- Unemployed
- Homemaker
- Student
- Retired
- Prefer not to answer

6. **6. What was your total household income before taxes during the past 12 months?** *
Mark only one oval.

- Less than $40,000
- $40,000 - $74,999
- $75,000 - $119,999
- $120,000 or more
- Prefer not to answer

7. **7. What is your age?** *
Mark only one oval.

- Below 18
- 18-34
- 35-49
- 50-64
- 65 or older
- Prefer not to answer

8. **8. What is your gender?** *

Mark only one oval.

- ( ) Female
- ( ) Male
- ( ) Transgender Female
- ( ) Transgender Male
- ( ) Gender Variant/Non-Conforming
- ( ) Other
- ( ) Prefer not to answer
